# The Clinical Significance of *Streptococcus mitis* Bacteremia

**DOI:** 10.1093/ofid/ofag588

**Published:** 2026-09-21

**Authors:** James H Acuff, Erin H Yang, Ananth Panchamukhi, Kenneth L Muldrew, Robert L Atmar, Daniel M Musher

**Affiliations:** Infectious Diseases Section, Department of Medicine, Baylor College of Medicine, Houston, Texas, USA; Division of Infectious Diseases, Department of Medicine, Massachusetts General Hospital, Boston, Massachusetts, USA; Baylor College of Medicine, Houston, Texas, USA; Department of Pathology and Immunology, Baylor College of Medicine, Houston, Texas, USA; Ben Taub Hospital, Harris Health System, Houston, Texas, USA; Infectious Diseases Section, Department of Medicine, Baylor College of Medicine, Houston, Texas, USA; Ben Taub Hospital, Harris Health System, Houston, Texas, USA; Infectious Diseases Section, Department of Medicine, Baylor College of Medicine, Houston, Texas, USA; Medical Care Line, Infectious Disease Section, Michael E. DeBakey Veterans Affairs Medical Center, Houston, Texas, USA

**Keywords:** bacteremia, *streptococcus mitis*, *streptococcus mitis* group, viridans streptococci

## Abstract

**Background:**

Except for their role in bacterial endocarditis, nonpneumococcal *Streptococcus mitis* group (SMG) bacteria have traditionally been regarded as commensals of the oropharynx. Widespread adoption of matrix-assisted laser desorption/ionization time-of-flight (MALDI-TOF) mass spectrometry has increased identification of these organisms and has led to increased concern about their clinical significance. We evaluated SMG bacteremia in a large multicenter cohort to characterize associated clinical syndromes and better define their clinical relevance.

**Methods:**

We performed a retrospective review of all adult patients from 2017 to 2025 who had SMG bacteremia at 3 hospitals within the Texas Medical Center. SMG bacteremia was defined as isolation of *S mitis*, *S mitis/oralis*, or *S mitis group* as determined by MALDI-TOF. Clinical syndromes were identified through chart review and assigned using consistent classification approaches informed by physician documentation, imaging, and microbiologic data.

**Results:**

Among 253 patients, neutropenia-associated bacteremia was most common (29.6%), followed by pneumonia (9.5%), infective endocarditis (8.7%), skin, soft tissue, or bone infections (7.9%), and other identified sources (12%). No clear source was identified in 9.5% of cases. Only 22.9% were classified as contaminants. High-grade bacteremia was common, with 47% of cases having ≥3 positive blood culture bottles. In contrast, patients in whom SMG was regarded as contaminant nearly always had only a single-bottle positive.

**Conclusions:**

*Streptococcus mitis* group bacteremia was associated with a broad spectrum of clinical syndromes, with most isolates being regarded as representing clinically significant infection.

## Teaser

In this multicenter retrospective cohort, nonpneumococcal Streptococcus mitis group bacteremia was associated with a broad spectrum of clinical syndromes beyond those traditionally recognized. Most cases were classified as clinically significant infection rather than presumed contamination, with blood culture positivity patterns strongly associated with this distinction.

Despite their remarkably close genetic relatedness to the important bacterial pathogen *Streptococcus pneumoniae*, members of the nonpneumococcal *Streptococcus mitis* group (SMG), particularly *S mitis, S infantis*, and *S oralis*, are often regarded as “commensal” or colonizing organisms [1], even though the use of the term “commensal” itself has been called into question [2], especially where these bacteria are concerned [3, 4]. An important recent review [5] stated that SMG are “nonpathogenic…although under immunocompromised conditions, some…may cause disease.” The widespread adoption of matrix-assisted laser desorption/ionization time-of-flight (MALDI-TOF) mass spectrometry in clinical microbiology laboratories has made it possible to distinguish these organisms from other alpha-hemolytic streptococci such as those in the *Streptococcus anginosus* (formerly *Streptococcus milleri*) group or ones that used to be called Group C or G streptococci. As a result, reports of SMG from clinical laboratories have become increasingly reliable, and their role as pathogens has become increasingly apparent.

Previous studies have implicated SMG as a cause of aspiration pneumonia [6], empyema [7], infective endocarditis [8], bacteremia in immunocompromised hosts [9–11], and silent bacteremia after dental procedures [12]. Most of these studies used traditional microbiology to identify the organism. A review of SMG infections in children [13] added a small number of cases of other clinical syndromes, including meningitis and sinusitis, although MALDI-TOF was not used for identification. We recently used quantitative bacteriology on high-quality sputum samples together with MALDI-TOF to show that viridans streptococci cause 18% of community-acquired pneumonia leading to hospitalization, with SMG predominating [14].

To our knowledge, a review of SMG infections in adults has not been reported for many years [15] and the clinical relevance of these organisms when isolated from blood cultures has not been well characterized. In an earlier report of *S mitis* bacteremia, about one-half of cases were attributed to endocarditis; the other one-half were dismissed as being clinically insignificant [16]. SMG comprised 22.8% of one well-characterized series of cases of viridans group streptococcal bacteremia [17]. We hypothesized that SMG isolated from blood cultures frequently represents clinically significant infection rather than contamination and is associated with a broader spectrum of clinical syndromes beyond its recognized association with infective endocarditis. Our objective was to characterize the clinical significance and spectrum of infections associated with SMG bacteremia among adults at 3 large academic hospitals.

## METHODS

### Study Design and Setting

We retrospectively reviewed medical records of patients who had an SMG bacterium (defined as *S mitis, S mitis/oralis*, or *S mitis* group) isolated from one or more blood cultures between 1 January 2017 and 30 June 2025 at Ben Taub Hospital (BTH), Lyndon B. Johnson Hospital (LBJH) and Michael E. DeBakey Veterans Administration Medical Center (MEDVAMC), all in Houston, Texas. BTH and LBJH are public hospitals within the Harris Health System (HH); these hospitals serve similar populations and data from them will be merged as HH. Patients were identified at HH using SlicerDicer (Epic Systems) and at MEDVAMC by TheraDoc®. Positive blood cultures were identified using conventional bacteriologic methods with organism identification performed by MALDI-TOF. Harris Health hospitals utilized BD BACTEC™ blood culture bottles, whereas MEDVAMC utilized BACT/ALERT® (bioMérieux) blood culture bottles. At all 3 hospitals, a standard blood culture set consisted of 8–10 mL of blood in each of 2 bottles, one aerobic and one anaerobic. Specimens were collected at the bedside via registered nurses or qualified patient care technicians following skin preparation with chlorhexidine gluconate and isopropyl alcohol.

### Data Collection and Case Definitions

Notes by primary care, specialty, and consulting physicians, laboratory data, results of imaging and discharge summaries were reviewed to characterize clinical syndromes and identify sources for the bacteremia. Fever was defined as body temperature >99.4 °F. Leukopenia and leukocytosis were designated as serum white blood cell (WBC) counts <3500 and >11 500 per mm^3^, respectively. Neutropenia was defined as <1000 neutrophils per mm^3^. Conventional definitions for disease states were used, and cases are described under these categories. Perhaps most difficult was to characterize bacteremia in cases without an apparent source and to distinguish these from contaminants. In such cases, we used clinical judgment to report “no source identified” or “suspected or presumed contaminant,” depending on the clinical context. When no source was identified in a neutropenic patient, infection was presumed to be present. Cases were identified as presumed contaminants if neutropenia was not present, no likely source was detected, a course of antibiotics was not given, or physicians treated despite their stated belief that the positive culture result reflected a contaminant.

### Statistical Analysis

Categorical variables were compared using chi-square or Fisher's exact tests, as appropriate. Continuous variables were compared using the Wilcoxon rank-sum test. Two-sided *P* values <.05 were considered statistically significant. Statistical analysis was performed using R version 4.6.0.

### Literature Review

We reviewed articles cited in PubMed under <*S mitis>, <S oralis>*, <*S infantis>* OR <viridans streptococcus> AND <bacteremia>, <infection>, OR <virulence>, excluding case reports. Whenever possible, we only included articles that used MALDI-TOF or molecular methods for identification [18, 19]. In accord with Kilian et al [20], we did not report on further speciation of these organisms unless a full genetic analysis was done.

## RESULTS

We identified 253 patients (index presentations) between 1 January 2017 and 30 June 2025 who had ≥1 blood culture(s) positive for SMG, 221 at HH and 32 at MEDVAMC (Figure 1). The median age of all patients was 58 (Table 1). Patients at HH were younger than those at MEDVAMC (median age 49 [IQR, 37–61] vs 71 [IQR 65.8–78.3] years, *P* < .001). Overall, 67.2% of patients were male, driven largely by the predominantly male MEDVAMC population. The peak temperature on the day the positive blood culture was obtained was >99.4 °F in 54.5% of patients. Neutropenia was more common at HH compared to MEDVAMC (72 of 221 [32.6%] vs 3 of 32 [9.4%] cases, *P* = .01), likely due to a greater proportion of patients with hematologic malignancy.

**Figure 1.**
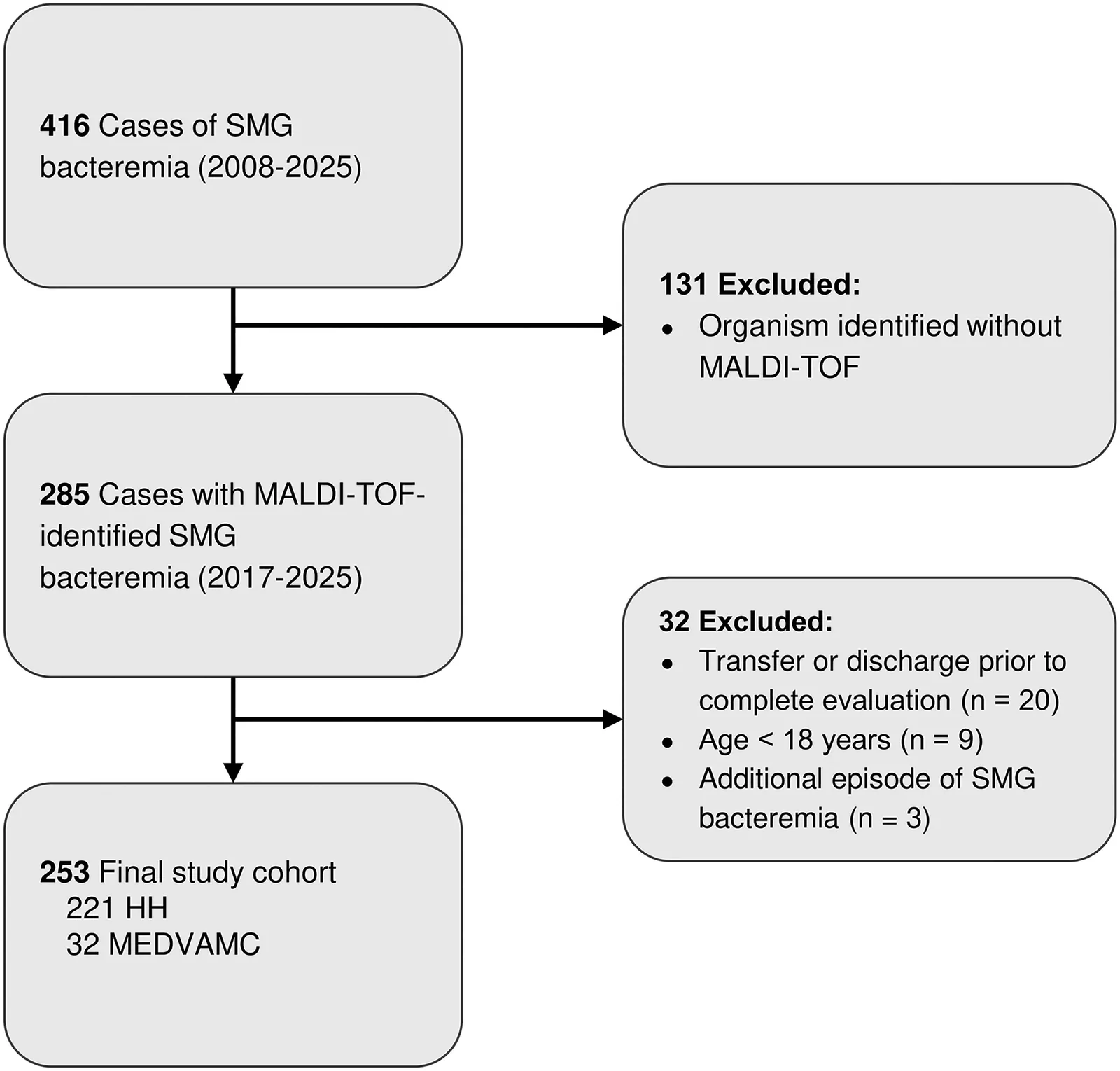
Study cohort selection flow diagram.

**Table 1.** Baseline Characteristics of Patients at Time of SMG Bacteremia (N = 253)

Characteristic	No. (%) or Value
**Hospital site**	
MEDVAMC	32 (12.6)
BTH	130 (51.4)
LBJH	91 (36.0)
**Demographics**	
Age, median (range), years	
Overall	58 (18–91)
MEDVAMC	71 (39–91)
Harris Health (HH)	49 (18–90)
Male sex	170 (67.2)
**Clinical characteristics**	
Temperature ≥99.5 °F^a^	138 (54.5)
White blood cell abnormalities	
Leukocytosis^b^	91 (36.0)
Leukopenia^c^	77 (30.4)
Neutropenia^d^	75 (29.6)

Abbreviations: BTH, Ben Taub Hospital; LBJH, Lyndon B. Johnson Hospital; MEDVAMC, Michael E. DeBakey VA Medical Center.

^a^Temperature at time of blood culture collection; threshold chosen to capture early or low-grade fever.

^b^WBC > 11 500.

^c^<3500 cells/µL; includes patients with neutropenia.

^d^ANC < 1000 cells/µL.

The number of blood culture bottles positive for *S mitis* impacted the certainty of diagnosis. In 119 of 253 (47.0%) cases, ≥3 bottles were positive (Figure 2); in 118 (99.2%) of these, a source for the bacteremia was identified or these patients were neutropenic. In contrast, in 50 of 58 (86.2%) cases in which only one bottle was positive, neutropenia was not present and no source was identified; these cases were classified as presumed contaminants (1/119 vs 50/58, *P* < .001). Additional clinical and microbiologic features distinguishing presumed contaminants from clinically significant cases are summarized in Table 2.

**Figure 2.**
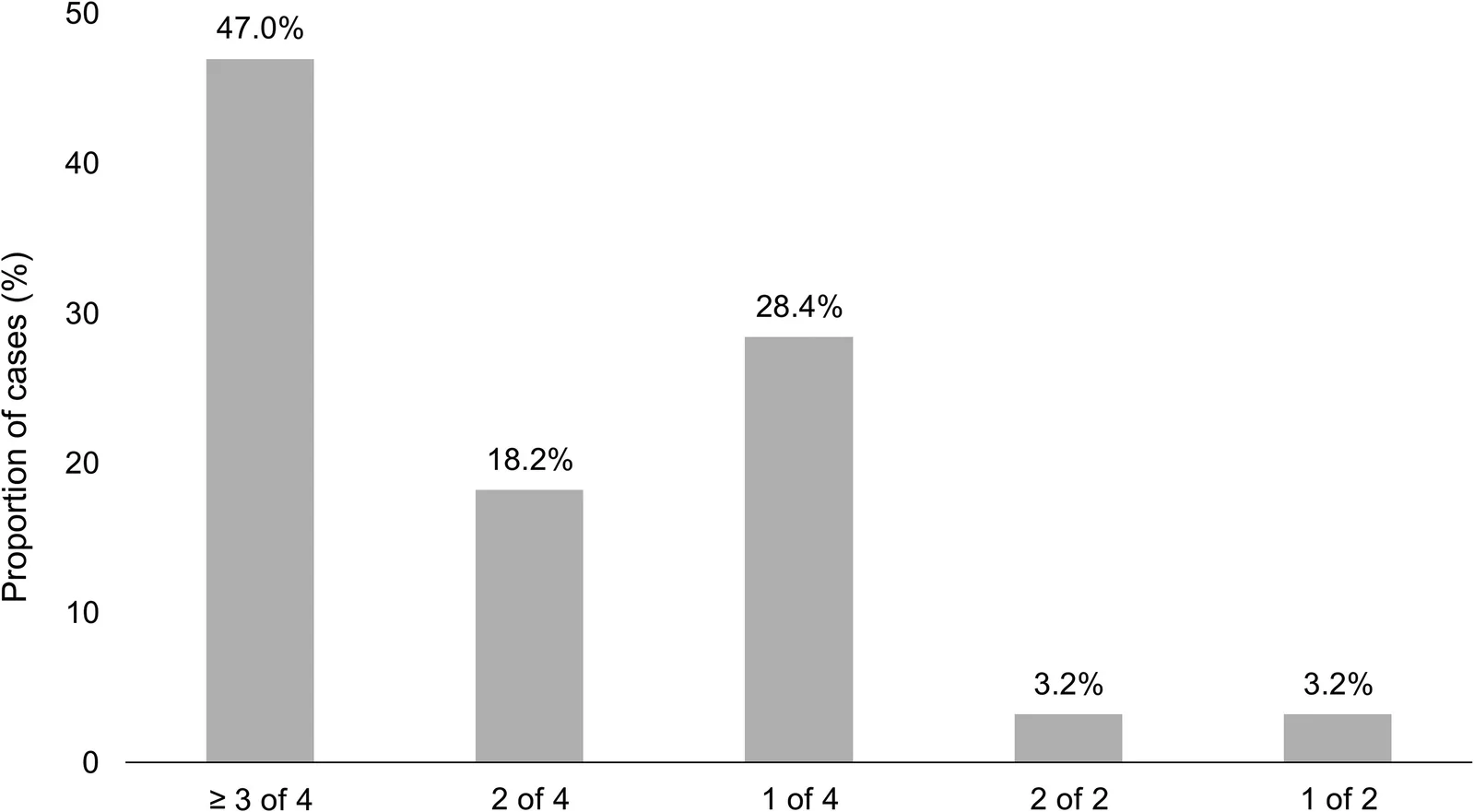
Blood culture positivity in *Streptococcus mitis* group (SMG) bacteremia.

**Table 2.** Clinical and Microbiologic Characteristics of Presumed Contaminant vs Clinically Significant Infection in SMG Bacteremia

Variable	Presumed Contaminant (n = 58)	Clinically Significant (n = 195)	*P* Value
Single-bottle positivity	50/58 (86.2%)	30/195 (15.4%)	<.001
High-grade bacteremia (≥3 of 4 bottles)	1/58 (1.7%)	118/195 (60.5%)	<.001
Polymicrobial bacteremia	29/58 (50%)	52/195 (26.7%)	<.001
Temperature ≥99.5 °F^a^	20/58 (34.5%)	118/195 (60.5%)	<.001
Temperature, median (IQR), °F	98.7 (98.0–100)	100.3 (98.3–102.5)	<.001

^a^Temperature at time of blood culture collection; threshold chosen to capture early or low-grade fever.

### Bacteremia With an Identified or Likely Source

One hundred twenty-seven of 253 (50.2%) patients with SMG bacteremia had an identified or likely source (Table 3).

**Table 3.** Clinical Syndromes Associated With SMG Bacteremia

Clinical Syndrome	No. (%)
**Pneumonia**	**24** (**9.5)**
Community-acquired pneumonia	11 (4.3)
HAP/VAP	2 (0.8)
Suspected aspiration pneumonia	11 (4.3)
**Infective endocarditis (IE)**	**22** (**8.7)**
Definite IE	17 (6.7)
Possible IE	5 (2.0)
**Skin, soft tissue, or bone infection**	**20** (**7.9)**
Diabetic foot infection	15 (5.9)
Cellulitis, osteomyelitis, other SSTI	5 (2.0)
**Intravascular device-associated**	**8** (**3.2)**
**Intraabdominal/genitourinary**	**7** (**2.8)**
**Dental or oropharyngeal infection**	**10** (**4.0)**
**≥2 likely sources**	**5** (**2.0)**
**No source identified**	**24** (**9.5)**
**Neutropenia-associated bacteremia**	**75** (**29.6)**
Likely source	31 (12.3)
No likely source	44 (17.4)
**Presumed contaminant**	**58** (**22.9)**
**Total**	**253** (**100)**

Abbreviations: HAP, hospital-acquired pneumonia; SSTI, skin and soft tissue infection; VAP, ventilator-associated pneumonia.

#### Infective Endocarditis

Twenty-two (8.7%) patients met the modified Duke criteria for “possible” or “definite” endocarditis [21]. The 5 patients who were classified as “possible” endocarditis were unable to undergo transesophageal echocardiography to confirm the presence of a vegetation due to the severity of clinical illness, and all received at least 6 weeks of antibiotics.

#### Pneumonia

Twenty-four (9.5%) patients had pneumonia, characterized as community-acquired or aspiration in 11 patients each and ventilator-associated in 2. No other bacterial pathogen was identified in respiratory secretions. Polymerase chain reaction (PCR) for a respiratory virus was done in 14 of these cases, with only one positive for influenza A. Pneumonia accounted for a significantly greater proportion of SMG bacteremia at MEDVAMC than at HH (10 of 32 [31.2%] vs 14 of 221 [6.3%], respectively, *P* < .001), consistent with greater age, debility, frequency of chronic aspiration, and chronic lung disease among older veteran patients.

#### Skin, Soft Tissue, or Bone Infection

In 20 (7.9%) patients, infection of skin, soft tissue, or bone was the source for the SMG bacteremia. These included 15 (75.0%) patients with diabetic foot infections, 3 (15.0%) patients with osteomyelitis, and 2 (10.0%) with cellulitis.

#### Intravascular

SMG bacteremia was attributed to an intravascular device in 8 (3.2%) cases. Devices included 5 central venous catheters, 1 peripherally inserted central catheter, 1 arteriovenous fistula, and 1 peripheral IV catheter.

#### Other

Other sources for bacteremia included dental or oropharyngeal infection (10 [4.0%] cases), an intraabdominal process, usually an abscess (7 [2.4%] cases), and multiple possible sources (5 [2.0%] cases).

#### Neutropenia With Identified or Likely Source

Thirty-one patients (12.3%) who were neutropenic at the time of blood culture collection had an identified or likely source of bacteremia. Source attribution in neutropenic patients is challenging because bacteremia frequently occurs in the setting of chemotherapy-associated injury of the mucosal barrier rather than a clearly localized infection. Commonly suspected sources include oropharyngeal pathology (stomatitis, mucositis, oral ulcerations), gastrointestinal pathology (enteritis, colitis, typhlitis), pulmonary infections, and intravascular device-associated infections.

### Bacteremia With No Identified Source

The largest category of 253 cases of SMG bacteremia and the most difficult to characterize consisted of 126 (49.8%) in which there was no recognized focus of infection.

#### Neutropenia

Of these 126 patients, 44 (34.9%) were neutropenic. Since blood cultures are only obtained in patients who are febrile or have other signs of infection, all were assumed to be infected and received antibiotic treatment; nearly all responded. Blood cultures were positive for additional organism(s) in 14 cases; all of these patients and many of those whose cultures were positive only for SMG received broad-spectrum antibiotics. Neutropenic patients who had SMG bacteremia were generally not evaluated for endocarditis. To the extent that endocarditis was sought, all studies were negative.

#### Non-neutropenic

Twenty-four of 126 (19.0%) patients with no recognized focus of infection who were not neutropenic were judged to be infected by treating clinicians and received ≥1 week of antibiotics even though no source was found.

#### Presumed Contaminants

SMG bacteremia was deemed to be clinically insignificant (presumed contaminants) in 58 (22.9%) cases. Most of these patients (79.3%) still received antibiotic treatment for SMG bacteremia, and none had any subsequent infection or complication that could be attributed to the bacteremia.

#### Polymicrobial Bacteremia

Bacteremia was polymicrobial in 84 of 253 (33.2%) patients. It was proportionally most common among intravascular device-associated infections (5 of 8, 62.5%), skin, soft tissue, or bone infections (12 of 20, 60.0%), and cases with multiple likely sources (3 of 5, 60.0%). Among patients whose SMG bacteremia was thought to result from contamination, 29 of 58 (50.0%) had polymicrobial blood cultures. The most common organisms in polymicrobial cases included coagulase-negative staphylococci (25.0%), *E coli* (15.5%), and *Klebsiella species* (8.3%).

#### Microbiology

Antimicrobial susceptibility testing was performed via MicroScan (Beckman Coulter) at MEDVAMC and BD Phoenix at HH. Of the 246 SMG isolates that underwent susceptibility testing, 71 (28.9%) showed intermediate susceptibility to penicillin. Only 6 showed intermediate susceptibility or resistance to ceftriaxone. The rest were susceptible to both drugs.

## DISCUSSION

*Streptococcus mitis* group, the predominant viridans streptococcus in the oropharynx [22], is also the streptococcus that is most likely to be identified in blood cultures. In one recent study [23], 40% of all streptococcal bacteremias at a large university hospital were due to SMG. Bacteremia with no apparent focus is regularly seen in other streptococcal infections including those due to groupable streptococci and *S pneumoniae* [24–26], so the absence of an identified source does not necessarily imply that the bacteremia was clinically unimportant. When severe immunocompromise and neutropenia are present, a focus of infection is usually not found, as seen in the present series.

Viridans streptococci have been increasingly well-documented to cause bacteremia in neutropenic hosts [9, 10, 27], in some cases associated with severe clinical disease or septic shock. At a major cancer center, Shelburne et al [9] found no source for *S mitis* bacteremia in 80% of cases, but the authors noted that if a patient with pneumonia had *S mitis* on sputum culture, it was not recorded because these organisms were regarded as “normal respiratory flora.” At another cancer center [11], over an 18-year period, 791 patients had viridans streptococci isolated from blood culture. Twenty (2.5%) of these were *S mitis*, and no source other than mucositis was mentioned. Importantly, in a study by Monk et al [10], among 210 patients with neutropenia and *S mitis* bacteremia, more than one-half had cardiac imaging without documentation of endocarditis in any case.

Osler recognized the importance of viridans streptococci as the cause of subacute bacterial endocarditis [28]. Among these organisms, *S mitis* is the most frequently isolated viridans streptococcus from blood cultures and is the most frequently implicated in endocarditis, although when bacteremia due to *Streptococcus mutans* or *Streptococcus sanguinis* does occur, it is substantially more likely than *S mitis* to be associated with endocarditis [8, 29, 30]. Seo et al [8] attributed only 7.6% of cases of *S mitis* bacteremia to endocarditis, compared to 31.3% of *S sanguinis* and 33.3% of *S mutans.* Consistent with Seo et al, we identified endocarditis in only 22 of 253 (8.7%) of our patients with *S mitis* bacteremia.

The initial adherence of bacteria to the heart valve, usually via surface-expressed proteins [31], is an important determinant of endocarditis [32]. The presence of a capsule probably inhibits such adherence, explaining the low incidence of endocarditis in pneumococcal bacteremia. Our recent demonstration of capsule on SMG organisms [33] may explain their less frequent role as a cause of endocarditis when compared, for example, to *S mutans*. Bacterial adhesion to intravascular devices, proliferation, and formation of biofilm has been amply documented since the seminal work of Costerton and Marrie et al [34], and an intravascular device was the source of SMG bacteremia in 8 (3.2%) of our cases.

Because of the prevalence of viridans streptococci in saliva, special techniques such as quantitative bacteriology in high-quality sputum specimens need to be used to convincingly identify them as a cause of pneumonia. Using such techniques in a prospective study, Musher et al [14] identified viridans streptococci as the primary cause in 18% and as a contributory cause, together with a coinfecting respiratory virus, in 8% of all cases of community-acquired pneumonia, with *S mitis* predominating; none of these patients was bacteremic. A subsequent study [33] showed that SMG, whether colonizing, causing pneumonia or isolated from blood, all have capsules, suggesting an important virulence mechanism by which these organisms may escape phagocytosis and cause pneumonia. Bell et al [35] implicated *S mitis* in empyema, and it is likely that some proportion of cases of empyema that, in the past, were attributed to “streptococci” [36] were also due to this organism.

In a 5-year regional study of 3931 infections of bones and joints [37], 93 (2.4%) involved streptococci of which 7 (7.5%) were due to *S mitis.* Presumed hematogenous (eg, vertebral osteomyelitis) and locally invasive (eg, osteomyelitis with and without orthopedic devices) sources were all implicated. Blood culture results were not specifically reported in these cases, although the overall rate of documented bacteremia in streptococcal bone and joint infections was only 14%. In a study of *Streptococcus tigurinis*, a subspecies of *S mitis,* Bourassa and Clarridge [38] attributed infections of natural or prosthetic joints to hematogenous spread from dental infections/procedures.

SMG cause other infections such as intraamniotic infection [39], spontaneous bacterial peritonitis [38], spinal epidural abscess [38], and infection of skin and soft tissues [17]. Associated bacteremia has only been rarely reported.

In our series, 97.2% of SMG isolates were studied for antibiotic susceptibility; 71.1% were fully susceptible to penicillin. The rest showed intermediate susceptibility. Some studies have found higher rates of penicillin non-susceptibility, especially in patients who have undergone treatment for malignancies [27].

Because SMG organisms have long been regarded as “commensals,” virulence factors have only recently been systematically studied. Although highly diverse by whole genome sequencing, *S mitis, S infantis,* and *S oralis* are closely related genetically to *S pneumoniae.* The presence of a capsule is the most prominent virulence factor in pneumococci, and nearly all SMG contain genes resembling those that encode capsule production in pneumococci [40–42]. The actual presence of capsules was, however, only recently identified by Musher et al [33] in 21 consecutive colonizing or disease-causing SMG isolates. Whole genome sequencing of SMG isolates has identified the variable presence of other genes encoding virulence factors of *S pneumoniae,* including pneumolysin, the principal pneumococcal disease-inducing substance [3], pneumococcal choline binding proteins such as *PsaA,* which mediates attachment to human tissues [43], proteases that degrade IgA, which may protect them against clearance by slgA [44], neuraminidase, which might increase susceptibility to coinfection with influenza virus [45], and *Lyt*A which encodes autolysin [46].

### Limitations

The retrospective nature of this study is a principal limitation. Clinical syndromes were assigned by the investigators utilizing diagnoses based on reanalysis of all data available in the electronic medical record but may be subject to misclassification bias, particularly in those cases with multiple or unclear sources for SMG bacteremia. Although MALDI-TOF is reliable, progressive refinement of reference databases during the study period may have influenced classification. Whole genome sequencing would have provided more accurate identification.

As noted above, bacteremia in neutropenic patients was classified as clinically significant, even when an infectious source was not identified. Although 71/75 (94.7%) of these cases had more than one blood culture bottle positive for SMG, contamination cannot be entirely excluded, particularly among the small number of patients with single-bottle positivity. Positive blood cultures in neutropenic patients are generally regarded as being clinically significant, particularly in our cohort in which cultures were obtained in the setting of inpatient fever. Nevertheless, it remains possible that our classification approach may have overestimated the proportion of clinically significant infection among neutropenic patients.

Patient demographics at the participating hospitals may also limit the generalizability of these findings. The population at MEDVAMC are generally older, male patients who may be more predisposed to certain clinical syndromes. Patients seeking care within HH, considered safety-net hospitals, may also not be representative of the average patient. Furthermore, availability of advanced diagnostic imaging (MRI, cardiac CT, and PET-CT), whether patient-dependent or institution-specific, may limit the ability to make definitive endpoint diagnoses.

## CONCLUSION

Despite belief to the contrary, SMG commonly cause infection with associated bacteremia, both in immunocompromised and non-immunocompromised hosts. In this multicenter retrospective evaluation, SMG bacteremia demonstrated substantial clinical heterogeneity and was most commonly associated with neutropenia-associated bacteremia, pneumonia, infective endocarditis, and skin and soft tissue infections. While some were identified as presumed contaminants, the great majority reflected clinically significant infection. These findings support the clinical significance of SMG organisms when isolated from blood cultures and highlight the importance of careful clinical assessment in such cases.
